# Optical and radiation shielding properties of PVC/BiVO_4_ nanocomposite

**DOI:** 10.1038/s41598-023-37692-y

**Published:** 2023-07-06

**Authors:** Said M. Kassem, M. I. A. Abdel Maksoud, Adel M. El Sayed, S. Ebraheem, A. I. Helal, Y. Y. Ebaid

**Affiliations:** 1 Radiation Protection and Dosimetry Department, National Center for Radiation Research and Technology (NCRRT), Egyptian Atomic Energy Authority (EAEA), Cairo, Egypt; 2 Radiation Physics Department, National Center for Radiation Research and Technology (NCRRT), Egyptian Atomic Energy Authority (EAEA), Cairo, Egypt; 3 Physics Department, Faculty of Science, Fayoum University, El Fayoum, 63514 Egypt; 4 Experimental Nuclear Physics Department, Nuclear Research Center (NRC), Egyptian Atomic Energy Authority (EAEA), Cairo, Egypt

**Keywords:** Engineering, Materials science, Nanoscience and technology, Optics and photonics, Physics

## Abstract

This study investigates the physical and optical properties as well as the radiation shielding capacity of polyvinyl chloride (PVC) loaded with x% of bismuth vanadate (BiVO_4_) (x = 0, 1, 3, and 6 wt%). As a non-toxic nanofiller, the designed materials are low-cost, flexible, and lightweight plastic to replace traditional lead, which is toxic and dense. XRD patterns and FTIR spectra demonstrated a successful fabrication and complexation of nanocomposite films. In addition, the particle size, morphology, and elemental composition of the BiVO_4_ nanofiller were demonstrated through the utilization of TEM, SEM, and EDX spectra. The MCNP5 simulation code assessed the gamma-ray shielding effectiveness of four PVC + x% BiVO_4_ nanocomposites. The obtained mass attenuation coefficient data of the developed nanocomposites were comparable to the theoretical calculation performed with Phy-X/PSD software. Moreover, the initial stage in the computation of various shielding parameters, such as half-value layer, tenth value layer, and mean free path, besides the simulation of linear attenuation coefficient. The transmission factor declines while radiation protection efficiency increases with an increase in the proportion of BiVO_4_ nanofiller. Further, the current investigation seeks to evaluate the thickness equivalent (X_eq_), effective atomic number (Z_eff_), and effective electron density (N_eff_) values as a function of the concentration of BiVO_4_ in a PVC matrix. The results obtained from the parameters indicate that incorporating BiVO_4_ into PVC can be an effective strategy for developing sustainable and lead-free polymer nanocomposites, with potential uses in radiation shielding applications.

## Introduction

Flexible lead-free radiation shielding nanocomposite films based on polyvinyl chloride (PVC) are a commonly used polymer combined with metal or metal oxide nanoparticles to create a radiation shielding layer within the film. These nanoparticles are used instead of lead as they offer comparable shielding properties without toxicity. The resulting films are flexible, lightweight, and cost-efficient. They can be used for various medical facilities, nuclear energy, aerospace, and industrial radiographic testing applications. However, it is important to note that the effectiveness of these films may vary depending on the type and intensity of radiation being shielded^1,2^. Because of its high density compared to other polymers, PVC can be an appropriate choice for producing composite materials for gamma ray shielding in the range of radio diagnostic energies by incorporating various nanoparticles (NPs)^1–6^.

Bismuth oxide Bi_2_O_3_ is a p-type semiconductor (with atomic number 83) that boasts a high density of 8.9 g/cm^3^. It is noteworthy that this material is non-toxic. It has also been found to possess gamma-ray shielding features equivalent to lead^7^. El-Sharkawy et al.^1^ have reported the incorporation of Bi_2_O_3_ NPs into recycled PVC as a potential solution for shielding gamma radiation. Also, Maksoud et al.^8^ intended to develop a novel gamma radiation shielding material based on a PVB (highly flexible, lightweight, lead-free) doped with bismuth oxide Bi_2_O_3_ and barium zirconate perovskite BaZrO_3_ as nano-metal oxides filler.

Vanadium pentoxide V_2_O_5_ is a material that shows considerable promise for use in microelectronic, electrochemical, and optical devices^9^. According to Hou et al.^10^, V_2_O_5_ provides photogenerated carriers and kinetic behavior and enhances a material's capacity to absorb ultraviolet radiation. Narayanan et al.^11^ reported that incorporating V_2_O_5_ into the polyaniline polymer matrix enhances the dielectric performance and electromagnetic shielding features of polymer nanocomposites while developing a highly effective composite network between V_2_O_5_ and polyaniline.

Bismuth vanadate (BiVO_4_)-based materials have been extensively employed in numerous applications as a significant ternary oxide semiconductor with a narrow band gap (2.2 eV)^12,13^.

This research aims to develop cost-effective, lead-free, eco-friendly, and sustainable PVC polymer nanocomposites reinforced by various proportions of BiVO_4_ nanofiller. Furthermore, this work investigates the structural, optical, and thermal characteristics of four produced PVC + x% BiVO_4_ nanocomposites to get insight into the influence of varied BiVO_4_ nanofiller loading on PVC performance. Further, the shielding parameters such as the half value layer (HVL), the effective atomic number Z_eff_, the effective electron density N_eff_, and the exposure buildup factor (EBF) were used to assess gamma ray interaction and penetration. The MAC produced by the MCNP5 simulation code was compared to those derived theoretically using the Phy-X/PSD database to assess the reliability of the findings acquired from the MCNP5 simulation.

## Materials and methods

### Materials

The materials utilized in this study include Poly (vinyl chloride) (PVC), a pure powder with a density of 1.4 g/mL at 25 °C manufactured by Sigma-Aldrich, USA. Also, bismuth oxide (Bi_2_O_3_, 99.9%) and vanadate pentoxide (V_2_O_5_, 99.7%) were procured from Alfa-Aesar, USA. Tetrahydrofuran (THF) was obtained from Aldrich, Germany.

### Synthesis of nanoparticles of BiVO_4_

The present study reports the successful synthesis of BiVO_4_ (BVO) using a solid-state reaction methodology. Bi_2_O_3_ and V_2_O_5_ were weighed by the stoichiometric 1:1 (Bi: V) molar ratio, serving as sources of Bi and V, respectively. The precursors were manually mixed using a pestle and mortar and subjected to ball milling for 1 h to achieve a uniform mixture. The resultant mixes were calcinated at 700 °C for 4 h.

### Preparation of PVC reinforced by BiVO_4_ nanocomposite films

**Figure 1:**
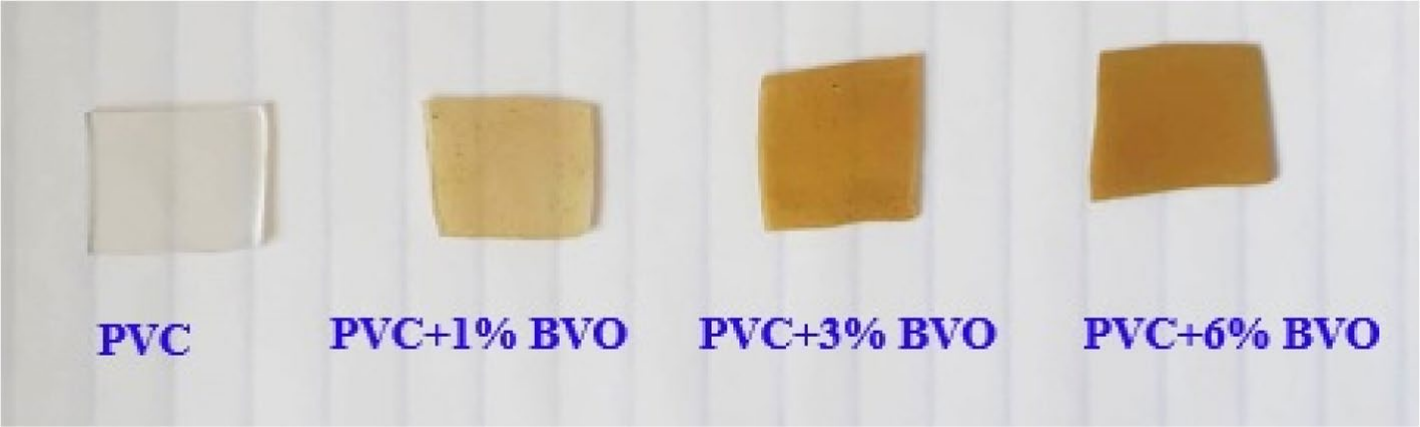
Photograph of the pure PVC and PVC + x% BVO nanocomposite films.

The BiVO_4_ nanopowder with varying weight percentages (x = 0, 1, 3, and 6 wt.%) was dispersed in THF using ultrasonic energy for 30 min. A PVC solution was formulated by dissolving 3.5 g of PVC powder in 100 ml of THF under continuous stirring for 2 h. Subsequently, the polyvinyl chloride (PVC) solution was mixed with the completely dispersed suspension of bismuth vanadate BVO nanofillers. The resultant mixture was subjected to continuous stirring for one hour. Subsequently, the generated suspension was carefully transferred into glass petri dishes and subjected to air drying. The PVC + x% BVO nanocomposite films were photographed as illustrated in Fig. 1, in addition to the weight fraction of the prepared PVC + x% BVO nanocomposite films shown in Table 1.

**Table 1:** Chemical composition (weight fraction), measured density, and heaviness for PVC + x% BVO nanocomposite films.

Sample code	Wieght fraction (wt%)		Density (ρ, g/cm^3^) ± 0.02	Heaviness (H, %) ± 0.2
	PVC	BiVO_4_ (BVO)		
PVC	100	0	1.448	12.75
PVC + 1%BVO	100	1%	1.545	13.62
PVC + 3%BVO	100	3%	1.652	14.55
PVC + 6%BVO	100	6%	1.875	16.51

### Characterization techniques

X-ray diffraction (XRD; Shimadzu XRD-6000) establishes the crystal structure of PVC + x% BVO nanocomposite films. HR-TEM analysis on a (JEOL-JEM-100 CX) was also used to investigate the shape and size of the synthesized BiVO_4_ powder nanoparticles. The PVC + x% BVO nanocomposite films are examined using the energy dispersive X-ray analysis spectra (EDAX), JEOL JSM-5600 LV, Japan. To corroborate the functional groups of the investigated films, Fourier transforms infrared (FT-IR) spectroscopy (NICOLET iS10 model instrument) is performed over a broad range (350–1800 cm^−1^) using a NICOLET iS10 model instrument. Using scanning electron microscopy (SEM, JEOL JSM-5500 LV, Japan), the morphology of the PVC + x% BVO nanocomposite films is determined. Using a double-beam spectrophotometer (Jasco, V-570 UV–VIS-NIR), the optical properties of PVC + x% BVO nanocomposite films in the 200–2500 nm wavelength range were evaluated.

By applying the following equation, the Archimedes rule was employed for calculating the density of toluene-soaked samples:

1 $$\rho = \frac{{W_{a} }}{{\left( {W_{a} - W_{T} } \right)}} \rho_{T}$$

where W_a_ is the sample's weight in the air, W_T_ is the nanocomposite film's weight in the toluene liquid, and ρ_T_ is the density of the toluene liquid (0.86 g/cm^3^ at ambient temperature).

It also shows the heaviness (H) of the samples that the following equation has calculated;

2 $$H \left( \% \right) = \left( { \frac{{\rho_{m} }}{{\rho_{Pb} }} } \right) \times 100$$

ρ_m_ is the prepared PVC composite film density, and ρ_Pb_ is the lead density.

### Radiation shielding theory

The present study employed the gamma-ray effectiveness relations as described below to evaluate the gamma-ray attenuation properties of polyvinyl chloride (PVC) containing different concentrations of bismuth vanadate (BVO) nanoparticles (NPs)^8,14–19^.

The present study involved the determination of the mass attenuation coefficient (MAC) and linear attenuation (LAC) of nanocomposite films composed of PVC and x% BVO. The NaI(Tl) scintillation detector (F4 tally) was utilized for this purpose, and the MCNP5 simulation code was employed.

3 $$I_{d} = I_{0} e^{ - LAC d}$$

4 $${\varvec{MAC}} = \left( {\frac{{{\varvec{LAC}}}}{{\varvec{\rho}}}} \right) = \user2{ }\frac{{{\varvec{ln}}\left( {\frac{{{\varvec{I}}_{0} }}{{{\varvec{I}}_{{\varvec{d}}} }}} \right)}}{{\user2{\rho d}}}\user2{ }$$

where I_o_ and I_d_ are the incidents and transmitted intensities.

The equation was applied by using the tables of Phy-X/PSD software for theoretical calculations:

5 $${\varvec{MAC}} = \left( {\frac{{{\varvec{LAC}}}}{{\varvec{\rho}}}} \right) = \mathop \sum \limits_{{\varvec{i}}} {\varvec{W}}_{{\user2{i }}} \left( {\frac{{{\varvec{LAC}}}}{{\varvec{\rho}}}} \right)_{{\varvec{i}}} \user2{ }$$

where W_i_ is the weight fraction of each i^th^ constituent in the sample.

Furthermore, measurements of LAC and MAC were used to achieve the MFP, Z_eff_, N_eff_, HVL, and TVL values according to the following relationships;

6 $${\varvec{MFP}} = \user2{ }\frac{1}{{{\varvec{LAC}}}}\user2{ }$$

7 $${\varvec{HVL}} = \user2{ }\frac{{{\varvec{ln}}\left( 2 \right)}}{{{\varvec{LAC}}}}\user2{ }$$

8 $${\varvec{TVL}} = \user2{ }\frac{{{\varvec{ln}}\left( {10} \right)}}{{{\varvec{LAC}}}}\user2{ }$$

Also, the present study involved the estimation of the transmission factor (TF), radiation protection efficiency (RPE), and a thickness equivalent to the lead thickness (X_eq_, cm) through the use of established relationships^20^;

9 $$TF \left( \% \right) = \frac{{I_{d} }}{{I_{0} }} \times 100$$

10 $$RPE \left( \% \right) = \left( {1 - \frac{{I_{d} }}{{I_{0} }}} \right) \times 100$$

11 $$X_{eq} = \frac{{LAC_{pb} }}{{LAC_{sample} }} \times t_{Pb}$$

where LAC_sample_ and LAC_Pb_ are the linear attenuation coefficient of the invesigated PVC nanocomposite films and lead respectively, and t_Pb_ is the thickness of the lead (cm).

The effective atomic number (Z_eff_) can be calculated by the following relation

12 $$Z_{eff} = \frac{{\mathop \sum \nolimits_{i} f_{i} A_{i} \left( {LAC} \right)}}{{\mathop \sum \nolimits_{j} \left( {\frac{{f_{j} A_{j} }}{{Z_{j} }}} \right)\left( {LAC} \right)}}$$

The effective electron density (N_eff_) can be estimated by the subsequent relation;

13 $$N_{eff} = \frac{{N_{A} }}{M} Z_{eff} \mathop \sum \limits_{i} n_{i}$$

where fi, Ai and Zj refer to the fractional prosperity, atomic weight and atomic number of the i^th^ constituent element, respectively and N_A_ represents the Avogadro constant.

## Results and discussion

### Characterization studies

**Figure 2:**
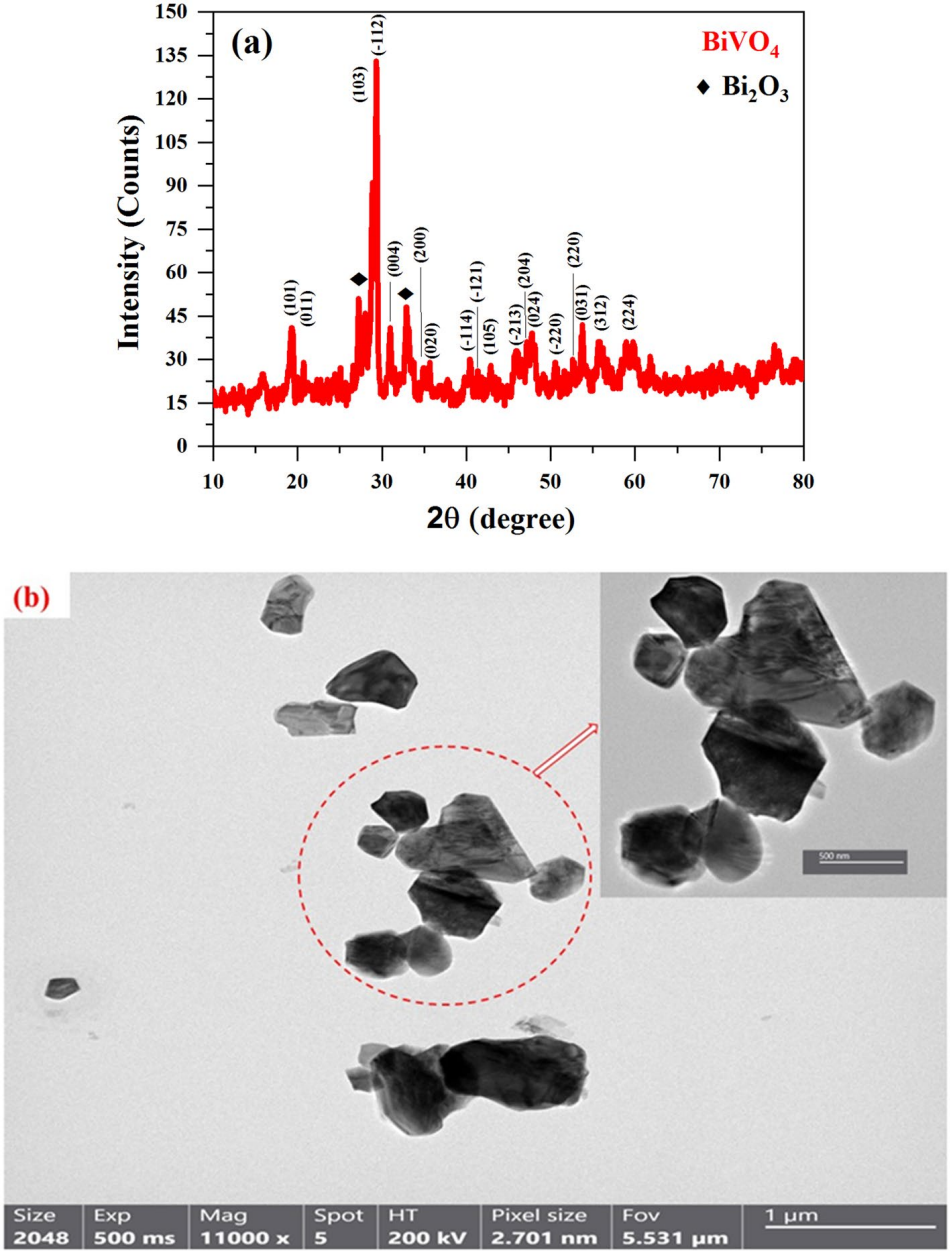
(**a**) XRD pattern, (**b**) TEM of BiVO_4_.

Figure 2a shows the XRD pattern of BiVO (BVO) nanopowder. The XRD pattern shows the formation of a pure BiVO_4_ phase with no impurity phases. The figure corresponds to the standard data for crystalline monoclinic BiVO (JCPDS card No.14-688) ^21,22^. In addition, it is possible to attribute the presence of minor peaks observed at 2θ = 27.21° and 32.91° to residual Bi_2_O_3_ resulting from the chemical synthesis procedure ^8^.

The crystallite size *D* was evaluated using the well-known Scherer equation:

14 $$D = \frac{{0.9 \times 0.154 \left( {{\text{nm}}} \right)}}{\beta \cos \left( \theta \right)}$$

where 0.154 nm is the wavelength (*λ*) of the used Cu *K*_α_ radiation, the calculated *D* value was 30.68 nm. Also, the TEM images show the asymmetrically arranged BiVO_4_ nanostructure, where shapes are plate-like, as illustrated in Fig. 2b.

**Figure 3:**
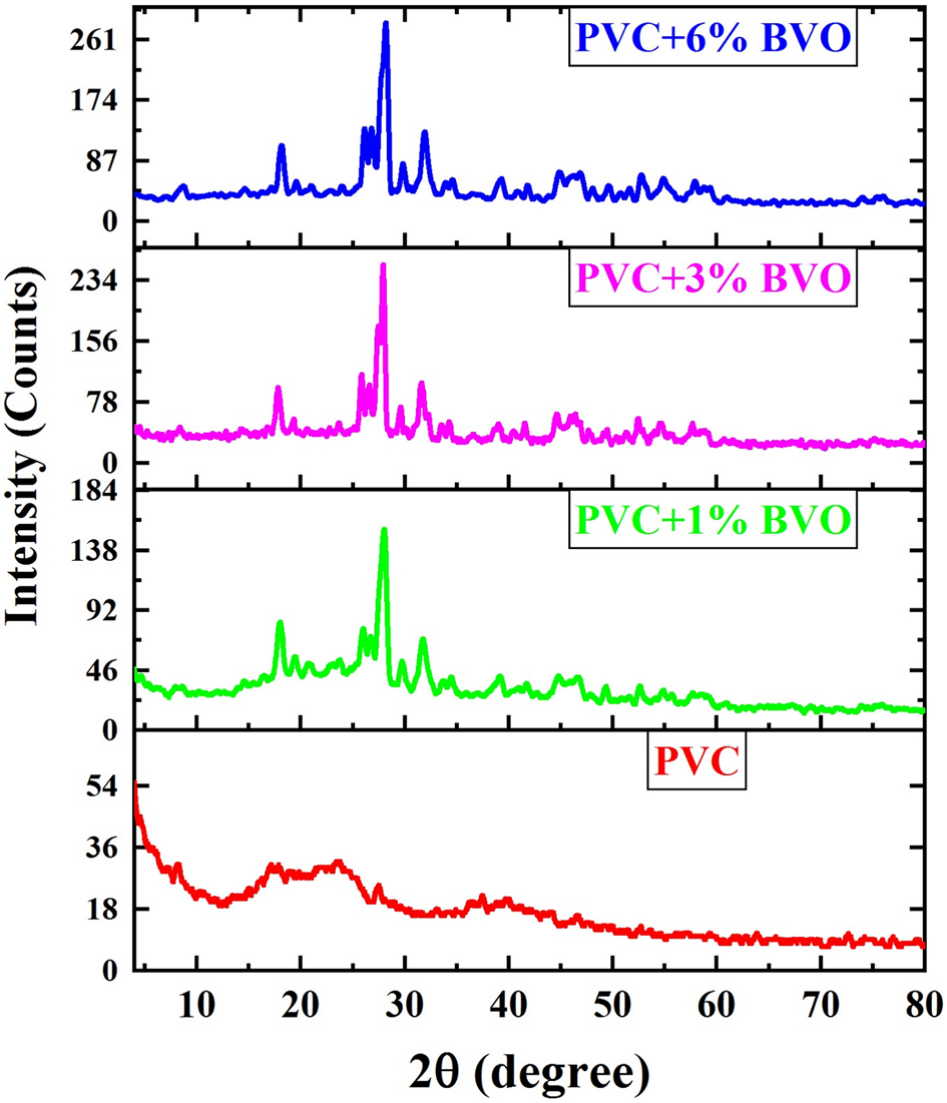
XRD patterns of PVC + x% BVO nanocomposite films.

Figure 3 depicts the X-ray diffraction patterns of fabricated PVC + x% BVO nanocomposite films with varying doping ratios of BiVO_4_. The observed patterns confirm the amorphous nature of the pure PVC polymer sample, as evidenced by the absence of distinct peaks in the characteristics of the amorphous phase. Moreover, depending on the weight percentage of the BVO nanofiller in the PVC, the intensity of the peaks for the nanocomposites varied and improved. This was detected even after 6% BVO nanofiller was added to PVC, showing successful preparation of PVC + x% BVO nanocomposite films, complexation, and uniform dispersion of BVO within the PVC polymer matrix^1,8^.

**Figure 4:**
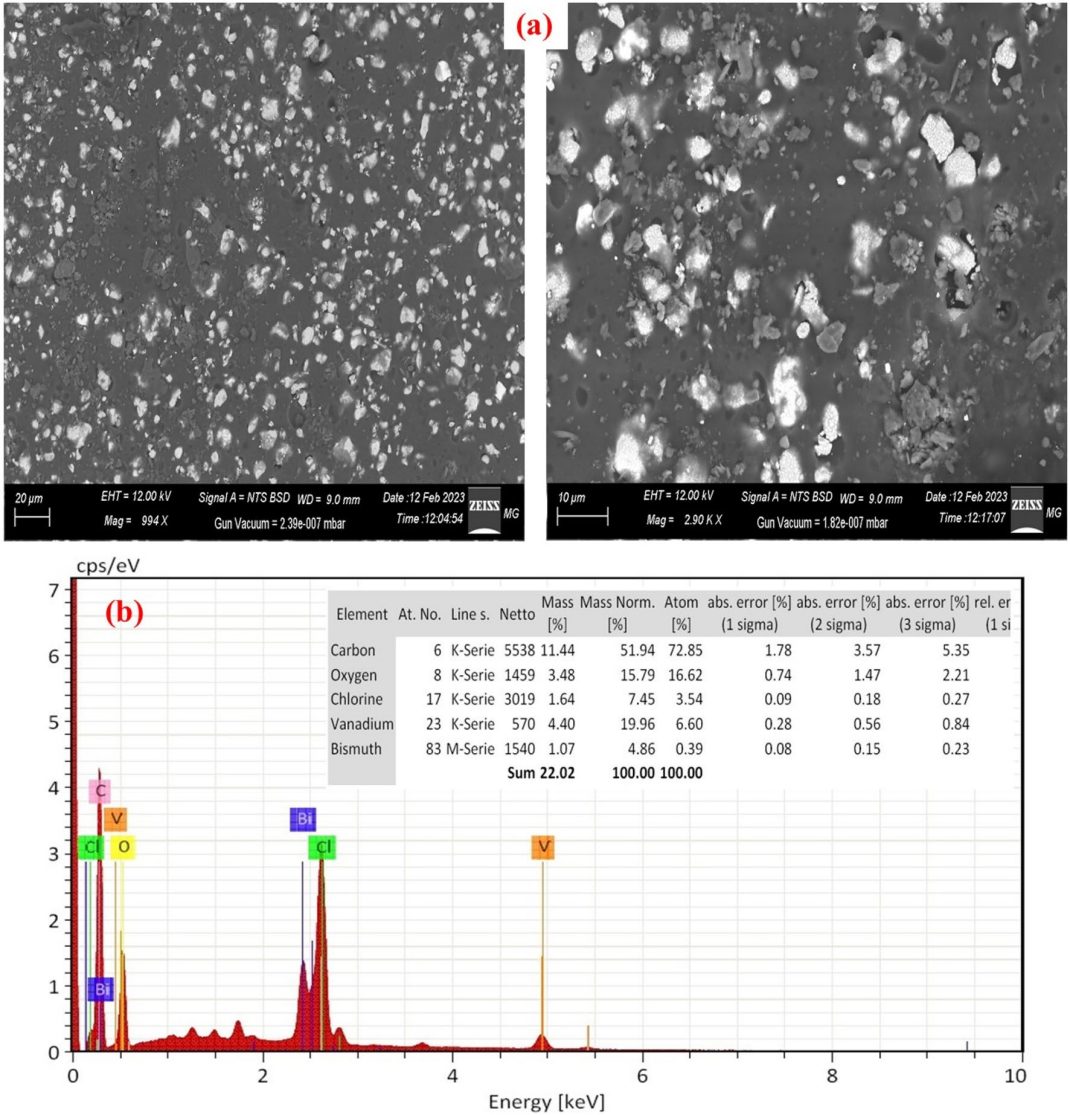
(**a**) SEM images, and (**b**) EDX of PVC + 6% BVO nanocomposite film.

Figure 4a illustrates the surface morphology characteristics of the nanocomposite films composed of PVC and x% BVO nanofiller. The SEM microstructure of the PVC + 6% BVO nanocomposite film, as depicted in Fig. 4a, displays a quasi-spherical and flakes structure due to the addition of BiVO_4_ nanofiller. The nanofiller is observed to be uniformly distributed throughout the PVC matrix with some minor agglomerations. The EDX spectra and elemental composition of the PVC + 6% BVO nanocomposite film depicted in Fig. 4b demonstrate the presence of signals attributed to bismuth, oxygen, and vanadate. This suggests that BiVO_4_ has been successfully incorporated into the PVC chains.

**Figure 5:**
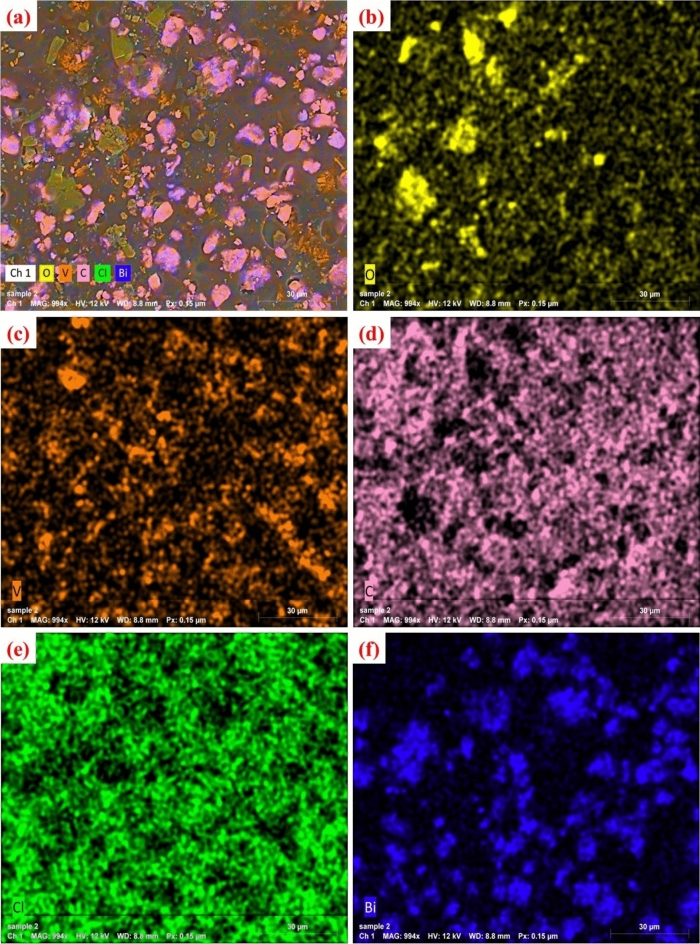
EDX mapping image for elemental composition of PVC + 6% BVO nanocomposite film.

Figure 5 depicts an elemental mapping analysis conducted on a nanocomposite film composed of PVC and 6% BVO. The BiVO_4_ nanofiller exhibits a uniform distribution, as evidenced by the yellow, orange, pink, green, and blue colors of O, V, C, Cl, and Bi, respectively. The uniform dispersion of BiVO_4_ nanofiller throughout the PVC matrix suggests a significant potential for attenuating low-energy gamma rays.

**Figure 6:**
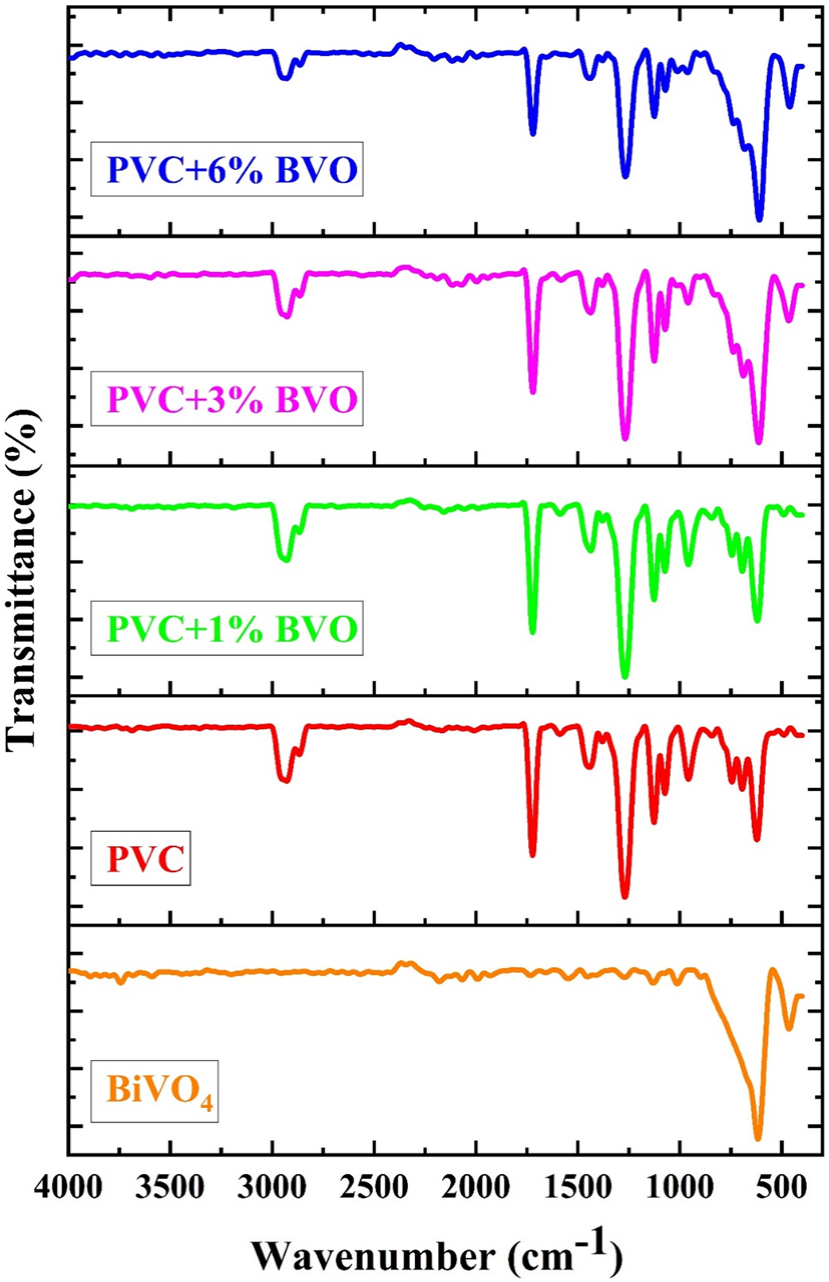
FTIR spectra of PVC + x % BVO nanocomposite films.

The Fourier transform infrared spectroscopic (FTIR) method was employed to detect and characterize the functional groups present in the prepared nanocomposite films. The spectral features of the nanocomposite films composed of PVC and x% BVO were analyzed within the range of 4000–400 cm^−1^, as depicted in Fig. 6. Based on the existing literature, the strong infrared band observed at 616.2 cm^−1^ can be attributed to the anti-symmetric stretching vibration arising from VO_4_. Additionally, the weak infrared band detected at 464 cm^−1^ is a consequence of the absorption of the Bi–O bond. The absorption band observed at 1012 cm^−1^, which exhibits high intensity, is attributed to the unshared stretching vibration of V–O. A vibration mode peak of V(Bi–O–Bi) is also observed at 1129.79 cm^−1^ ^23,24^. The present study reports the observation of characteristic absorption peaks for pure PVC. Specifically, the CH_2_ deformation mode was observed at 1269 cm^−1^, the –CH stretching mode at 2928 cm^−1^, the CH_3_ stretching mode at 1440 cm^−1^, the C–H rocking mode at 1269 cm^−1^, the trans-C–H wagging mode at 957 cm^−1^, the C^_^Cl stretching mode at 743 cm^−1^, and the cis CH-wagging mode at 620 cm^−1^ and 693 cm^−1^. These findings are consistent with a previous report. The introduction of BVO fillers resulted in a new peak at 609 cm^−1^ in the growth of the cis CH wagging peak. An increase in BVO nanofiller loading increased the intensity of the 464.15 cm^−1^ peak. The bands observed at 2178.8 cm^−1^ and 1722 cm^−1^ may be attributed to the ion–dipole interactions of the BVO fillers, the aromatic rings present in the styrene molecules, or the –CH_2_– (methylene) units in PVC molecules^1,25^.

### Optical spectroscopy analysis

**Figure 7:**
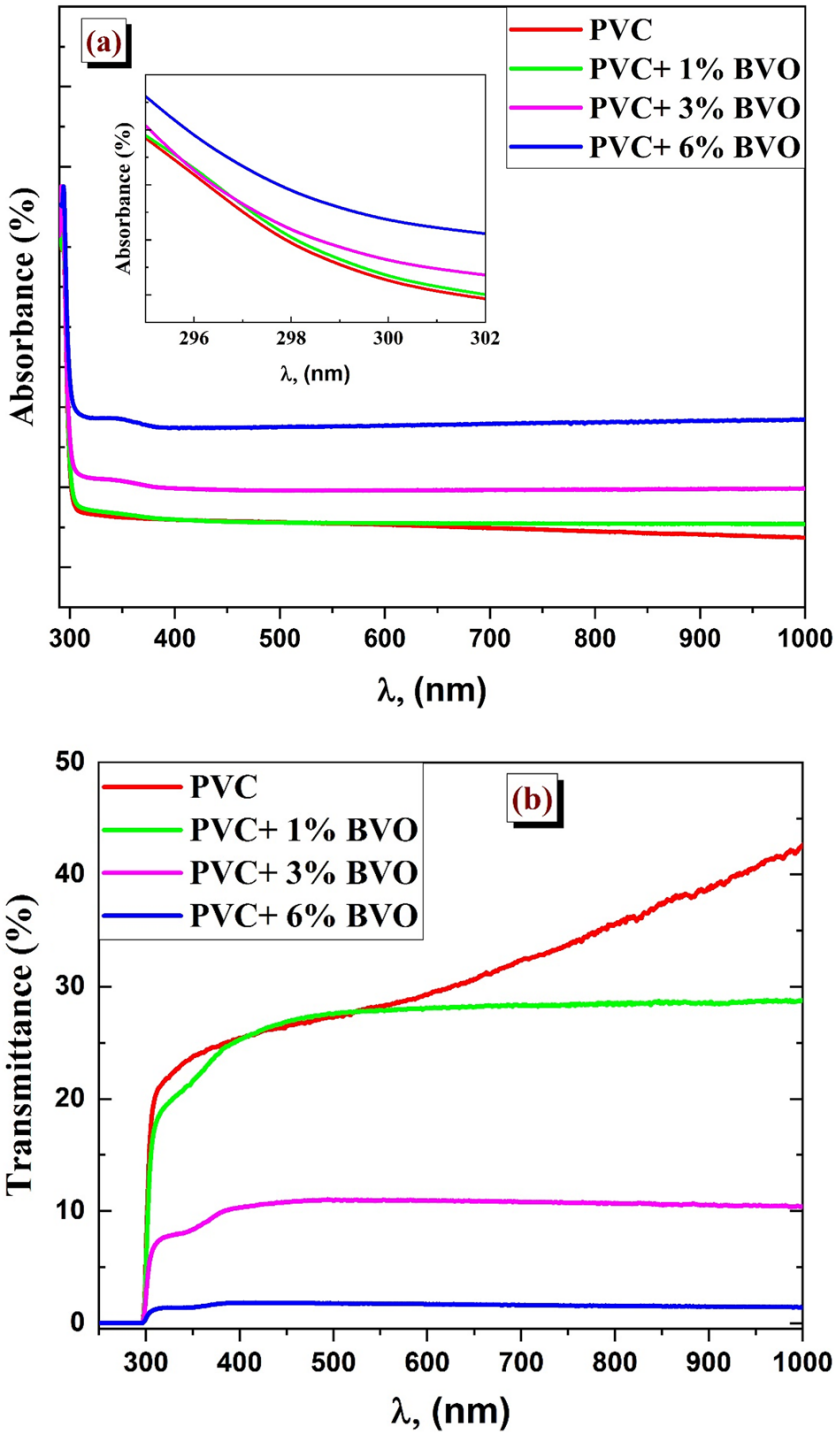
UV–vis spectra of PVC + x % BVO nanocomposite films: (**a**) absorbance (A%) and (**b**) transmittance (T*%*).

Figure 7 depicts the absorption and transmittance spectra of a PVC + x% BVO nanocomposite film produced within the UV–Vis wavelength range of 200–1100 nm. Figure 7a demonstrates the absorption spectra, showing that the absorption of PVC + x% BVO nanocomposite films improves with an increase in the concentration of BiVO_4_ nanofillers. Incorporating BiVO_4_ in the PVC matrix has resulted in a broad near-visible band in the absorbance spectra. This can be attributed to the increased surface area of the blend due to the doping agent, which in turn provides more possibilities for light rays to be absorbed. The broad spectral band in close range exhibits a nearly saturated tendency towards longer wavelengths due to complexation between the polymer and BiVO_4_ filler. The observed absorption peaks could be attributed to the electronic transition between the valence and conduction bands within the host lattice. The transmittance spectra of a nanocomposite film consisting of PVC and x% BVO are depicted in Fig. 7b. The pure PVC matrix exhibits a high level of transparency, exceeding 90% in the visible spectrum. Notably, incorporating BiVO_4_ nanofillers into PVC has yielded a rough surface, leading to a significant increase in absorption and a corresponding decrease in transmittance of PVC with increasing concentration of BiVO_4_ nanofillers. The findings strongly correlate with the FTIR measurements^26,27^.

It is notable for evaluating the E_g_ of the PVC + x% BVO nanocomposite films using the previously mentioned expression.

15 $$\alpha h\nu = \, {\mathbf{B}}\left( {h\nu - E_{opt} } \right)^{s}$$

The previous formula pertains to determining the optical energy band gap, wherein B is a constant, s denotes either 1/2 or 2 for allowed direct and indirect transitions, respectively, and Eopt represents the corresponding energy band gap.

**Figure 8:**
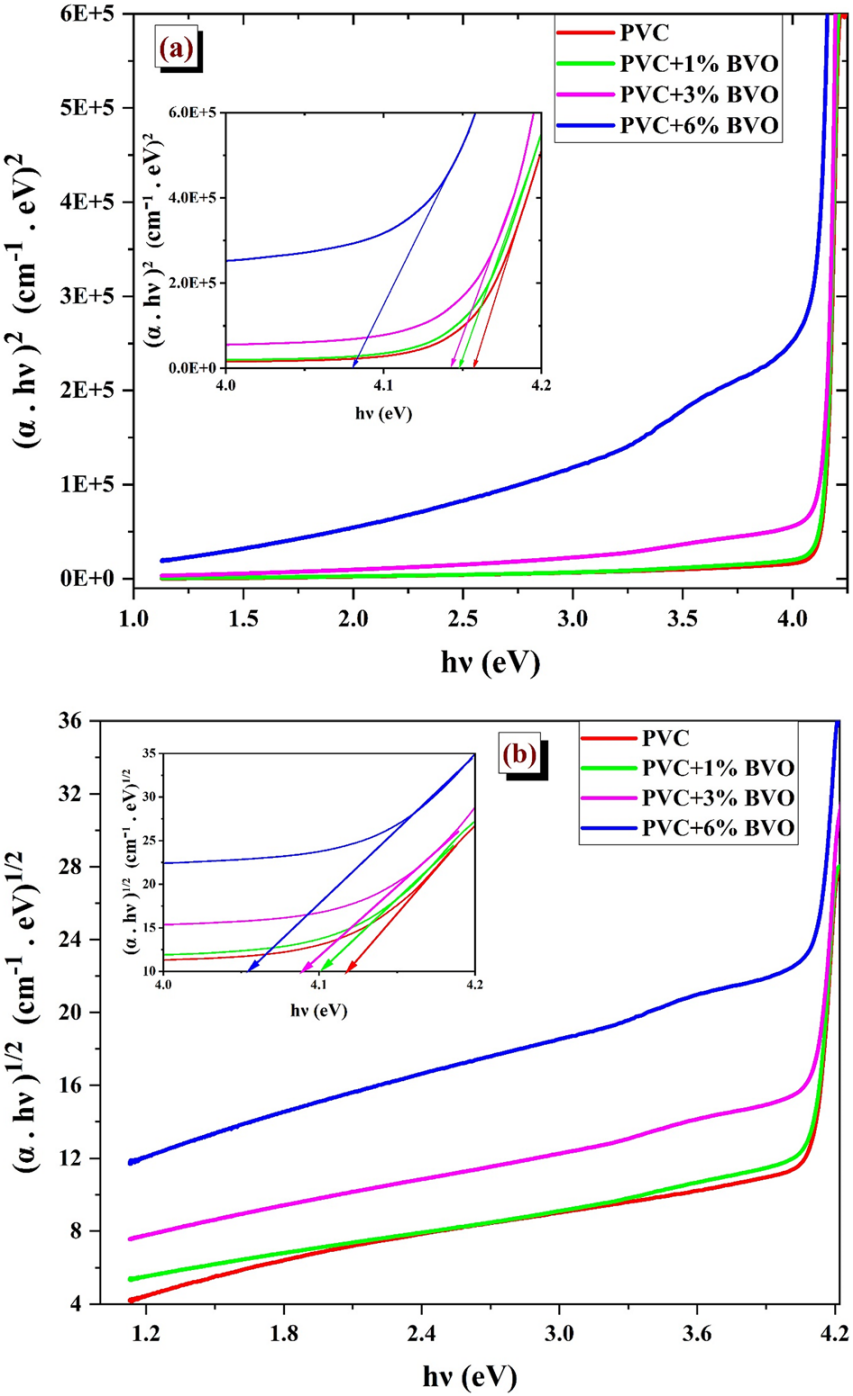
Variation of (**a**) (αhυ)^2^ and (**b**) (αhυ)^1/2^ with (hυ) of PVC + x % BVO nanocomposites.

Figure 8 illustrates the variations of direct (αhυ)^2^ and indirect (αhυ)^1/2^ transition regarding the (hυ) of PVC + x % BVO nanocomposite films. The Tauc plot was utilized to determine the energy required to stimulate an electron from the valence band toward the conduction band. The values obtained for PVC were 4.11 eV ($${E}_{g}^{dir})$$ and 4.142 eV ($${E}_{g}^{ind})$$, while those for PVC + 6% BVO nanocomposite film were reduced to 4.01 eV ($${E}_{g}^{dir})$$ and 4.06 eV ($${E}_{g}^{ind})$$, as presented in Table 2. These results are consistent with previous investigations^2^.

**Table 2:** Optical energy band gap, Urbach energy of PVC + x % BVO nanocomposite films.

Sample code	E_gap_ (eV)		E_Urbach_ (meV)
	Direct	Indirect	
PVC	4.12	4.16	78
PVC + 1% BVO	4.10	4.15	81
PVC + 3% BVO	4.09	4.13	117
PVC + 6% BVO	4.05	4.08	170

Using the following empirical formula, the Urbach energy (Eu) has to be estimated to evaluate the level of disorder in the films under consideration, which is caused by the development of localized states within the forbidden band gap^8,15^:

16 $${\varvec{\alpha}} = {\varvec{\alpha}}_{0} + \user2{ exp}^{{\left( {\frac{{\user2{h\upsilon }}}{{{\varvec{E}}_{{\varvec{u}}} }}} \right)}} \user2{ }$$

The symbol (αo) represents a constant value. The Urbach energy values of the PVC + x % BVO nanocomposite films can be established by estimating the reciprocal slope of the linear sections of ln(α) versus (hυ). The estimated values of E_u_ energy for these films can be found in Table 2. The investigation reveals that the values of E_u_ exhibited an increase from 78 meV in the pure PVC matrix to 170 meV in the PVC + 6%BVO nanocomposite film, which was doped with a high concentration of BiVO_4_ nanofillers. The present findings validate that introducing BiVO_4_ nanofillers in PVC polymer matrix results in a corresponding increase in band tail energy, indicating a heightened concentration of localized states^2^.

### Radiation attenuation parameters

**Figure 9:**
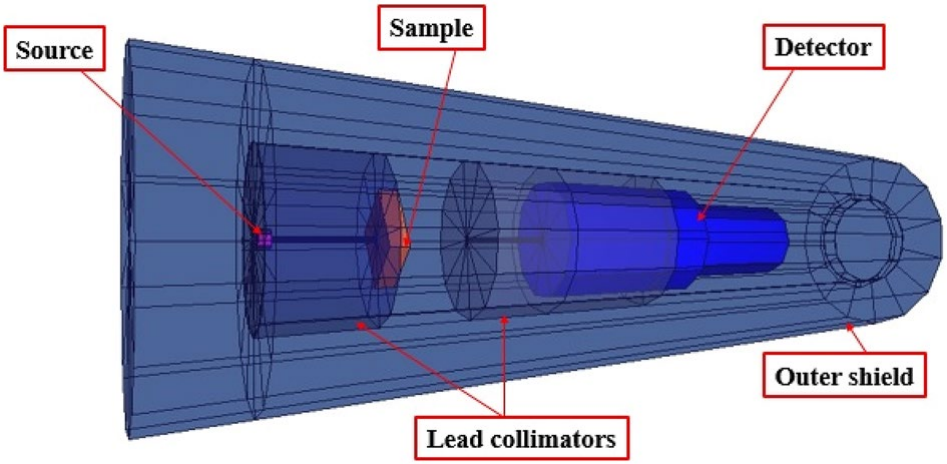
MCNP simulation geometry.

The present study uses a Monte Carlo N-particle transport code to simulate the transportation of electrons, neutrons, gamma rays, X-rays, and all their combinations. The MCNP5 simulation code is a radiation transport code developed and produced by the Los Alamos National Laboratory (LANL)^28,29^. To execute an MCNP simulation, the MCNP input file must contain accurate data on the geometry, source card, and composition (material card). The input file utilized in the MCNP code emulates the geometrical and compositional attributes of the configuration depicted in Fig. 9. The NaI(Tl) scintillation detector (F4 tally), lead collimator, materials that were being studied, and lead-surrounded shield have been defined in the cell and surface cards concerning their density values, dimensions, and geometric shapes. According to the (SDEF) source card definition, the point source is a radioactive source whose position, investigated energies, and emission direction are specified. The simulation was conducted utilizing 10^6^ particles using an NPS card. The Monte Carlo N-Particle (MCNP) simulation yields a relative error below 1%. The present study has evaluated the shielding efficacy of nanocomposite films composed of PVC and x% BVO for various energies of Ba-133, Eu-152, Co-60, and Cs-137. The computations were performed using a recently developed online tool, Phy-X/PSD ^30^.

In accordance with the data presented in Table 1, the PVC + 6%BVO composite exhibits heaviness among the composites studied while still being 83.48% lighter than lead. It is noted that fabricated polymer composites have applaudable weightlessness when compared with traditional shielding materials such as lead ^31^. In particular, the density enhances from 1.448 for PVC bland to 1.875 g/cm^3^ for PVC + 6%BVO film. The observed trend in the examined PVC + x% BVO nanocomposite films indicates an increase in density with an increase in BiVO_4_ nanofiller. The total density of a particle-filled composite can be determined by applying the rule of mixture, which establishes a relationship between the densities of the composite's constituent particles. The observed rise in the magnitude can be ascribed to the comparatively greater density of BiVO_4_, which is found at 6.98 g/cm^3^, in contrast to the density of PVC, which is recorded at 1.448 g/cm^3^ ^32^.

**Figure 10:**
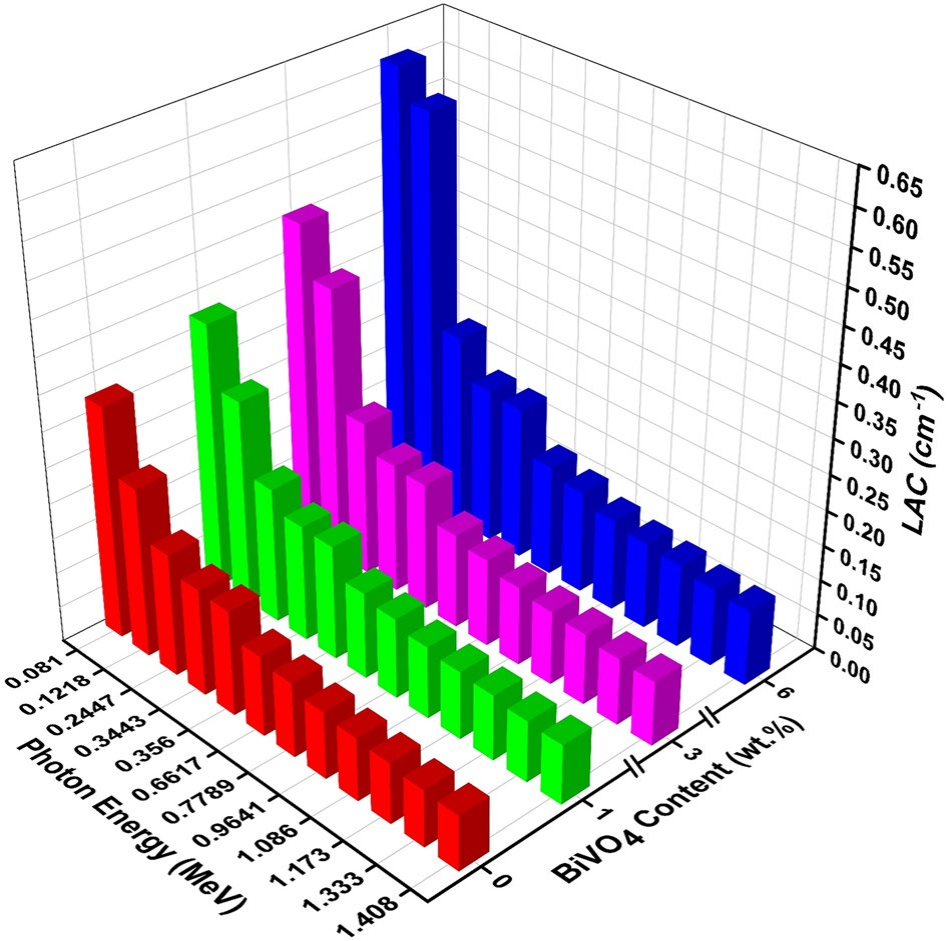
The variation of LAC of PVC films as a function of the BiVO_4_ content in the energy range 0.081–1.408 MeV.

Figure 10 depicts the LAC (Linear Attenuation Coefficient) values for PVC composite samples concerning the BiVO_4_ content and various photon energies extending from 0.081 to 1.408 MeV. The rise in BiVO_4_ content results in a noticeable growth in the linear attenuation coefficient (LAC) due to incorporating BiVO_4_ powder in PVC, which impacts the absorption of gamma rays. The incorporation of x% BVO into PVC nanocomposite films results in an increase in both the molecular weight and density of the material. Generally, the linear attenuation coefficient (LAC) tends to decrease as the energy level grows. This can be attributed to the heightened ability of high-energy photons to penetrate through materials. The data indicates that the linear attenuation coefficient (LAC) of pure polyvinyl chloride (PVC) exhibits a decreasing trend as the energy of the incident gamma rays increases. The LAC values exhibit a tendency to rise as the BVO ratio rises, with the lowest (highest) value of 0.3292 cm^−1^ (0.0783 cm^−1^) at 0.081 MeV (1.408 MeV) for pure PVC, and the highest (lowest) value of 0.6141 cm^−1^ (0.1073 cm^−1^) at 0.081 MeV (1.408 MeV) for PVC + 6% BVO nanocomposite film. The values of the PVC + x% BVO nanocomposite films' LAC were influenced by two distinct parameters: the energy of photons and the ratio of BiVO_4_ doping. Figure 10 demonstrates a trend of exponential reduction in the LAC values as the energy of incident gamma photons improves. The observed behavior can be attributed to the three primary photonic interactions with matter, which have been previously examined^33^. The probabilistic nature of the interaction between matter and gamma photons is a well-established behavior^1,2^.

**Figure 11:**
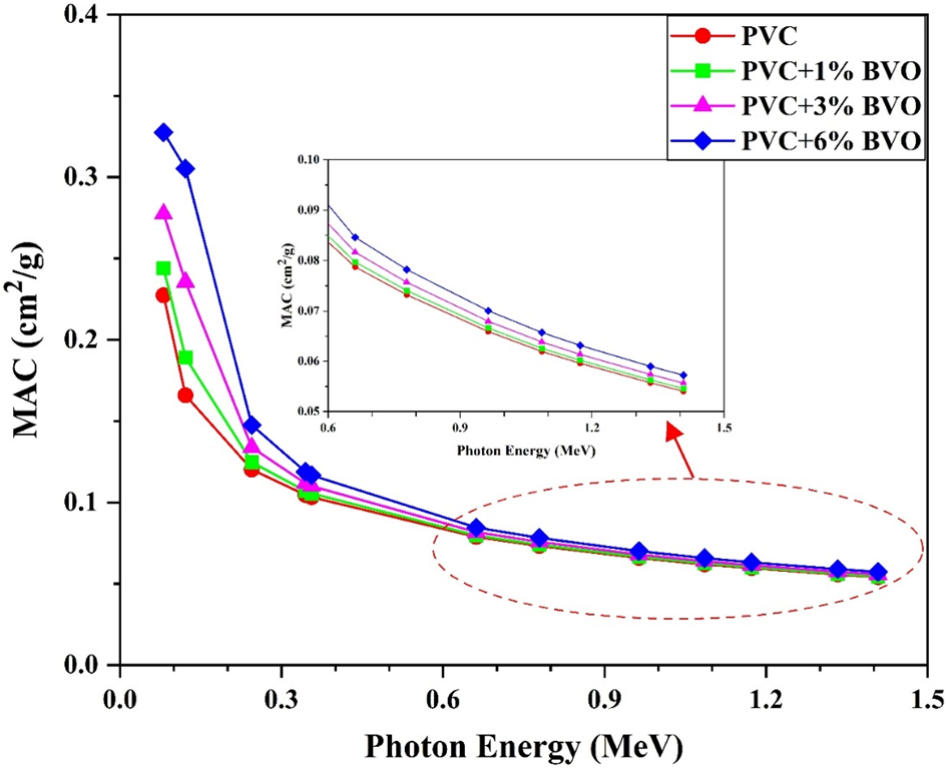
The variation of MAC of PVC + x% BVO nanocomposite films.

Determining a material's shielding effectiveness involves measuring its mass attenuation coefficient, which is a crucial parameter in the context of gamma-ray shielding. As mentioned above, the parameter quantifies the proportion of gamma-ray photons that go through attenuation while crossing the PVC + x% BVO nanocomposite attenuator. Figure 11 illustrates the MAC values of PVC (polyvinyl chloride) composite samples concerning the BVO content in conjunction with the corresponding data for all photon energies. The study observed a significant decrease in the MAC values of PVC + x% BVO nanocomposites alongside an increase in gamma-radiation energy for certain weight fractions of BiVO_4_ nanofillers. The correlation between MAC and the energy of gamma-ray photons has become dependent upon the associated partial photon interactions. The energy characteristics of MAC may be elucidated by considering the relative significance of all these partial photon processes^34^.

The probabilistic variance regarding the interaction between the gamma photons and matter was examined across three distinct energy regions, including the low energy range marked by photoelectric interaction (PE). The current research demonstrates that the absorption cross-section is directly proportional to the fourth or fifth power of atomic numbers (Z^4^ or Z^5^) and inversely proportional to the photon energy (E^(7/2)^) of the constituent materials inside the nanocomposite structure. For energy levels below 0.2 MeV, it can be observed that all samples exhibit their maximum MAC values. At photon energies exceeding 0.5 MeV, the Compton Scattering (CS) phenomenon becomes the most prevalent type of interaction. This interaction is characterized by a proportional relationship between the cross-section and both the atomic number (Z) and photon energy (E), leading to a reduction in the variability of the mass attenuation coefficient (MAC) values. The MAC values gradually declined in response to the rising energy levels within the specified region. At energy levels exceeding 1 MeV of gamma-radiation, the dominant interaction has become pair production (PP). It is observed that there exists a proportional correlation between the interaction cross-section and both Z^2^ and log E^33^.

At an energy level of 81 keV, the PVC + 6% BVO composite film exhibited a maximum value of 0.3275 cm^2^/g for MAC, while the pure PVC film showed a maximum value of 0.2273 cm^2^/g. The minimum values of MAC, which were determined at 1408 keV, amounted to 0.0572 cm^2^/g (PVC + 6% BVO) and 0.05407 cm^2^/g (PVC).

Table 3 and Fig. 11 demonstrate a consistent rise in MAC with the increase of the weight percentage of BiVO_4_ nanofillers in the produced nanocomposites, as observed across all gamma-ray photon energies examined. The observed phenomenon could be attributed to the homogeneous dispersion of BiVO_4_ particles, which has a high atomic number and density. This property enhances the probability of interaction between the nanocomposite shield and gamma-ray photons, leading to energy transfer between them. The same behavior of conduct has been observed in earlier studies^2,35,36^. Theoretical MAC was calculated utilizing the online Phy-X/PSD tool. The simulated outcomes of the MAC for PVC films blended with x% of BVO NPs were found to be analogous to those obtained through the use of Phy-X/PSD. This indicates that the values for the MAC presented in Table 3 are consistent.

$${{\varvec{\Delta}}}\left( \% \right) \, = \left| {\left( {\left( {{\mathbf{MCNP}}} \right){-}\left( {{\mathbf{Phy}} - {\mathbf{X}}} \right)} \right)/\left( {{\mathbf{MCNP}}} \right)} \right|*{\mathbf{100}}$$

**Table 3:** Mass attenuation coefficients for the PVC + x% BVO nanocomposite films.

Mass attenuation coefficient (cm^2^/g)												
Energy (MeV)	PVC			PVC + 1% BVO			PVC + 3% BVO			PVC + 6% BVO		
	MCNP	Phy-X	Δ (%)	MCNP	Phy-X	Δ (%)	MCNP	Phy-X	Δ (%)	MCNP	Phy-X	Δ (%)
0.081	0.2273	0.2269	0.213	0.2440	0.2436	0.169	0.2774	0.2772	0.096	0.3275	0.3275	0.014
0.1218	0.1660	0.1657	0.122	0.1892	0.1889	0.124	0.2356	0.2353	0.127	0.3052	0.3048	0.130
0.2447	0.1202	0.1205	0.245	0.1247	0.1250	0.261	0.1338	0.1342	0.290	0.1475	0.1480	0.327
0.3443	0.1046	0.1049	0.306	0.1070	0.1073	0.317	0.1117	0.1121	0.337	0.1189	0.1193	0.364
0.356	0.1032	0.1035	0.269	0.1055	0.1058	0.281	0.1100	0.1103	0.302	0.1167	0.1171	0.331
0.6617	0.0787	0.0794	0.811	0.0797	0.0804	0.816	0.0817	0.0823	0.824	0.0846	0.0853	0.835
0.7789	0.0732	0.0736	0.584	0.0740	0.0745	0.591	0.0757	0.0762	0.605	0.0782	0.0787	0.624
0.9641	0.0659	0.0665	0.923	0.0666	0.0672	0.929	0.0680	0.0686	0.938	0.0700	0.0707	0.952
1.086	0.0619	0.0627	1.248	0.0626	0.0634	1.252	0.0638	0.0646	1.258	0.0657	0.0666	1.267
1.173	0.0596	0.0603	1.222	0.0602	0.0609	1.226	0.0614	0.0621	1.234	0.0632	0.0640	1.245
1.333	0.0557	0.0565	1.416	0.0563	0.0571	1.419	0.0574	0.0582	1.426	0.0590	0.0598	1.435
1.408	0.0541	0.0550	1.642	0.0546	0.0555	1.644	0.0557	0.0566	1.648	0.0572	0.0582	1.655

Additional parameters that can be employed to demonstrate a material's photon shielding capacity include the Half-value layer (HVL) and tenth-value layer (TVL). The HVL (Half-Value Layer) and TVL (Tenth-Value Layer) are frequently utilized to measure nuclear shielding effectiveness. These measures are based on the principle that as the thickness of a shield increases, the level of radiation passing through it decreases. Specifically, the HVL is defined as the thickness of a shield required to reduce the radiation level by one-half, while the TVL is the thickness required to reduce the radiation level by one-tenth of its original value. The mean free path (MFP) quantifies the typical distance traversed by a photon before interacting with a shielding material^33^.

**Figure 12:**
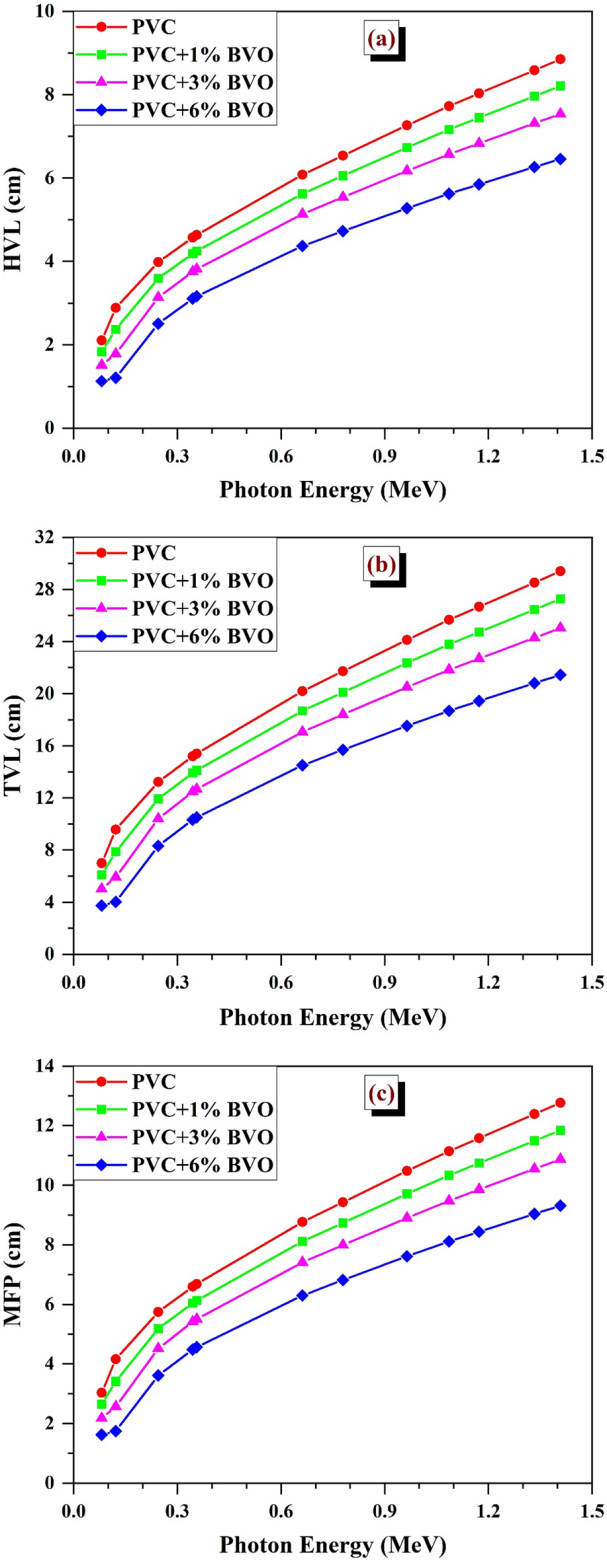
Variation of (**a**) HVL, (**b**) TVL, and (**c**) MFP values for PVC + x% BVO nanocomposite films with selected gamma-ray energies.

The study involved the computation of HVL, TVL, and MFP for PVC + x% BVO nanocomposites using gamma-photon energies ranging from 0.081 to 1.408 MeV. The analysis outcomes are presented in Fig. 12. Typically, the variables exhibit a positive correlation, varying in the same direction, either increasing or decreasing simultaneously. The efficiency of gamma-ray attenuation is directly proportional to the values of HVL, MFP, and TVL at different photon energies. Lower values of these parameters indicate a greater probability of photon interaction with the shielding material, resulting in more efficient attenuation^1,16,33^.

The HVL values of PVC + x% BVO nanocomposite films were observed to be at their maximum (smallest) at 1.408 MeV (0.081 MeV), with corresponding values of 8.852 (2.106), 8.211 (1.837), 7.539 (1.512), and 6.459 (1.29) cm for x = 0, 1, 3, and 6%, respectively, as depicted in Fig. 12a.

Tenth-Value Layer (TVL) values are shown in Fig. 12b. Here, the values measured at the minimum gamma ray energy (0.081 MeV) were 6.994 cm, 6.103 cm, 5.024 cm, and 3.749 cm for x = 0, 1, 3, and 6%, respectively. At the highest energy level of gamma rays examined (1.408 MeV), the TVL measurements obtained were 29.407 cm, 27.278 cm, 25.044 cm, and 21.457 cm for x values of 0, 1, 3, and 6%, respectively.

The graphic illustration in Fig. 12c demonstrates a correlation between MFP and photon energy, indicating that the former is contingent upon the latter. The findings indicate that the amount of BiVO_4_ nanofillers rising enhances the shielding of gamma-radiation within the energy range of 0.081 to 1.408 MeV. Based on the recorded values at 0.081 MeV (1.408 MeV), the measurements yielded results of 3.038 cm (12.771 cm), 2.6504 cm (11.846 cm), 2.182 cm (10.877 cm), and 1.628 cm (9.319 cm) for x = 0, 1, 3, and 6%, respectively.

Thus, it is apparent from Fig. 12 that the half value layer (HVL), tenth value layer (TVL), and mean free path (MFP) values are significantly influenced by the composition of each nanocomposite material in the shielding material and the energy of the gamma rays.

**Figure 13:**
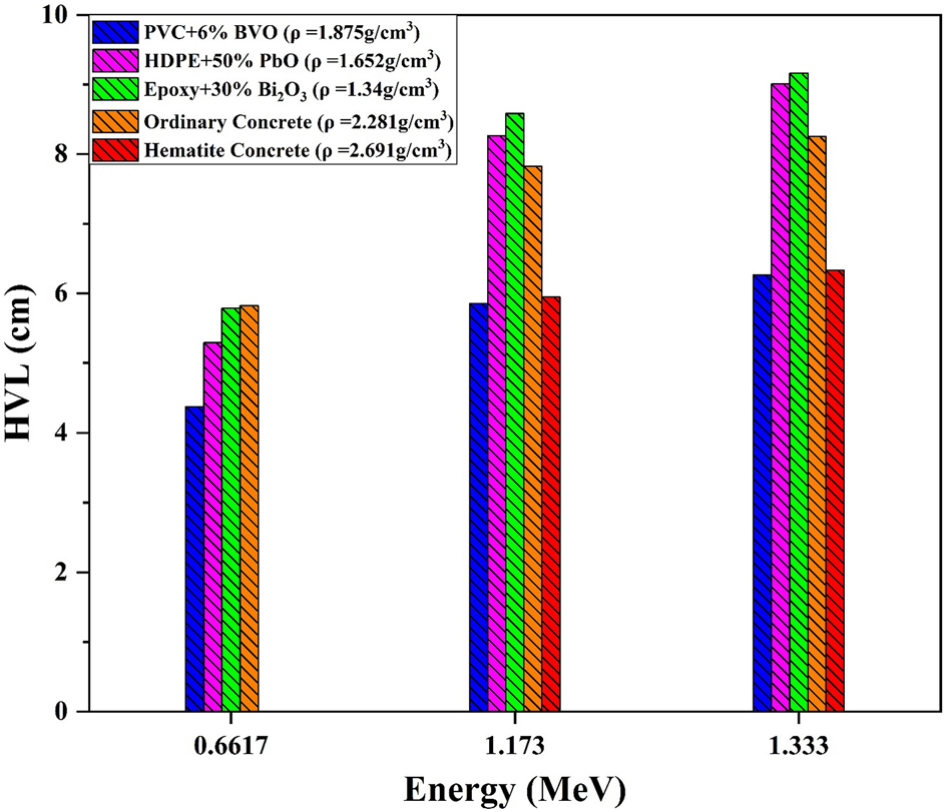
Variation of HVL as a function of photon energy for the PVC + 6% BVO nanocomposite films in comparison to standered shielding materials.

Figure 13 showed that the HVL of PVC + 6% BVO nanocomposite film in comparable to other privous works. Also, the HVL values of the PVC + 6% BVO (ρ = 1.875 g/cm^3^) nanocomposite film reported against energies 0.662, 1.173, and 1.333 MeV are lower than the HVL values of the epoxy + 30% Bi_2_O_3_ composite (ρ = 1.34 g/cm^3^), HDPE + 50%PbO NPs (ρ = 1.652 g/cm^3^), ordinary concrete (ρ = 2.281 g/cm^3^), and hematite concrete (ρ = 2.691 g/cm^3^) that variants due to different density and content composite of compared shield materials. It is indicated their impact on radiation attenuation parameters ^32,37–39^.

The transmission factor (TF) is a metric that quantifies the ratio of the number of photons that have penetrated a given material (Np) to the total number of photons that have been incident upon it (Ni). On the other hand, the radiation protection efficiency (RPE, %) is an indicator of the proportion of photons that have been absorbed (Na) within PVC + x% BVO nanocomposite films to the total number of incident photons (Ni).

**Figure 14:**
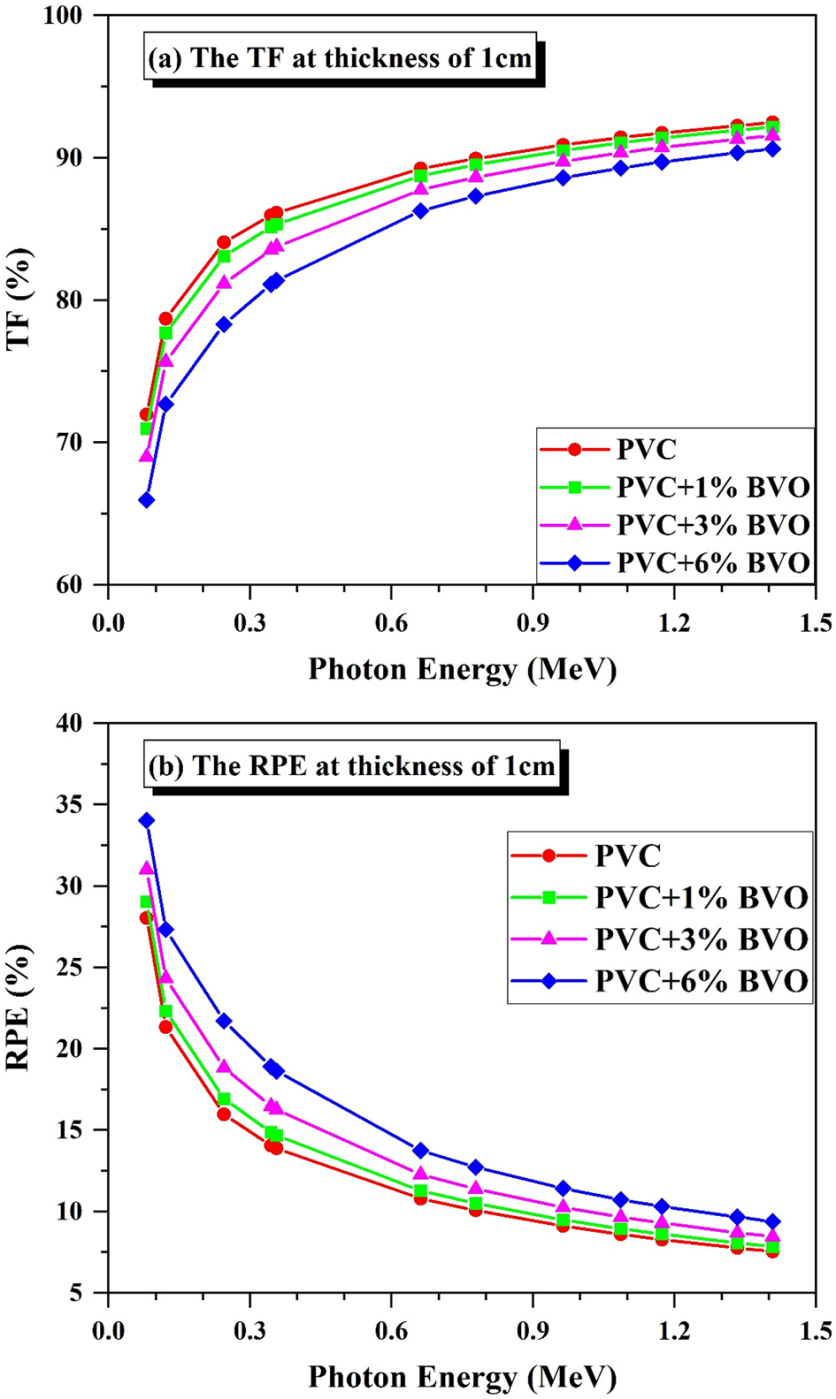
Variation of (**a**) Transmission factor (TF, %) (**b**) Radiation protection efficiency (RPE, %) versus the incident gamma-ray energy.

The impact of high photon energy on the transmission factor (TF) and radiation protection efficiency (RPE) of PVC + x% BVO nanocomposite films at a sample distance of 1 cm is depicted in Fig. 14. As per the findings presented in Fig. 14a, the transmission factor (TF) exhibits a consistent rise with an increase in photon energy. At the same time, it displays a decline with an increase in the proportion of BiVO_4_ nanofiller. The TFs at 0.081 MeV and 1.408 MeV for PVC, PVC + 1% BVO, PVC + 3% BVO, and PVC + 6% BVO are approximately 71.97% and 92.47%, 70.97% and 92.17%, 68.97% and 91.55%, and 65.97% and 90.63%, respectively, with the lowest and highest values. Figure 14b illustrates a notable reduction in the RPEs of the PVC + x% BVO nanocomposite films at 1 cm thickness of the analyzed samples as the photon energy increases, while it rises with a higher proportion of BiVO_4_ nanofiller. The RPEs with the highest and lowest values are observed to be around 28.03% and 7.53%, 29.03% and 7.83%, 31.03% and 8.45%, and 34.03% and 9.37% at energy levels of 0.081 MeV and 1.408 MeV for PVC, PVC with 1% BVO, PVC with 3% BVO, and PVC with 6% BVO, respectively.

**Figure 15:**
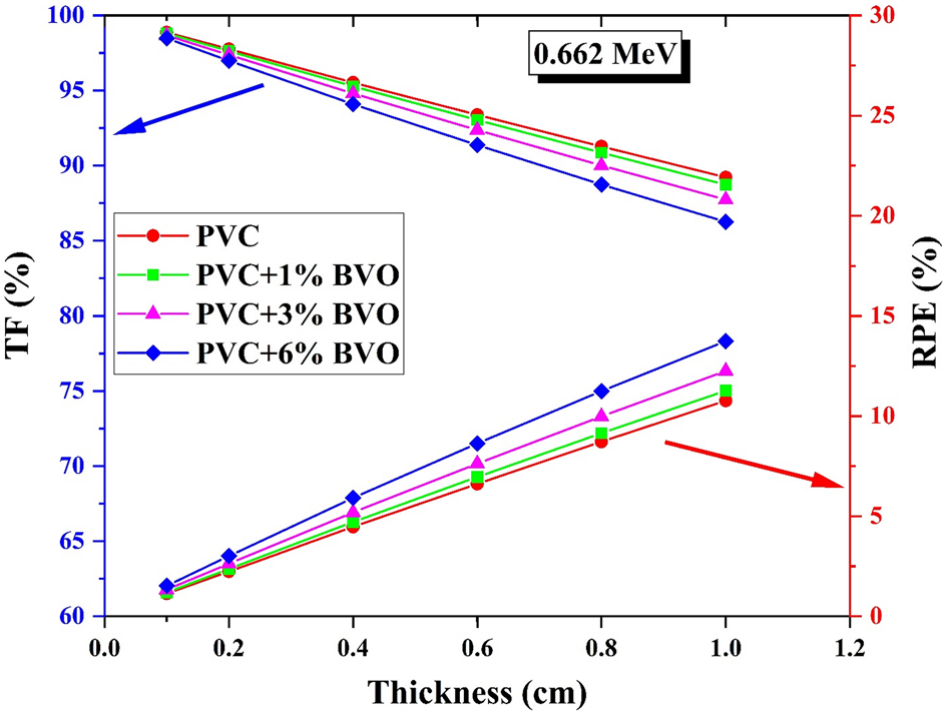
Relation between the (TF, %) and the (RPE, %) versus PVC + x% BVO nanocomposite films thickness at 0.662 MeV energy.

Figure 15 illustrates the changes in TF and RPE concerning the thickness of the PVC polymer nanocomposites under investigation at 0.662 MeV. The results indicate a decrease in TF and an increase in RPE as the thickness of the PVC + x% BVO nanocomposite films increases. The results indicate that the TF values decreased in pure PVC and PVC + 6% BVO samples. Specifically, the TF values decreased from 98.87 to 89.23% for pure PVC and from 98.47% to 86.26% for PVC + 6% BVO when the thickness of the tested samples was increased between 0.1 and 1 cm. Contrary to this, the RPE demonstrates an increase of 1.13% to 10.77% for pure PVC and 1.53% to 13.74% for PVC with 6% BVO when the thickness of the tested samples is increased between 0.1 and 1 cm. Increased amounts of BiVO_4_ nanofiller enhance the molar mass and density of the produced PVC + x% BVO nanocomposite films. There is an increase in the number of absorbed photons (Na) and a decrease in the number of penetrating photons (Np) when the frequency of collisions between the generated photons and the electrons in the material rises. Consequently, there was a reduction in the (Np/Ni) ratio associated with an increase in the (Na/Ni) ratio^40–42^.

**Figure 16:**
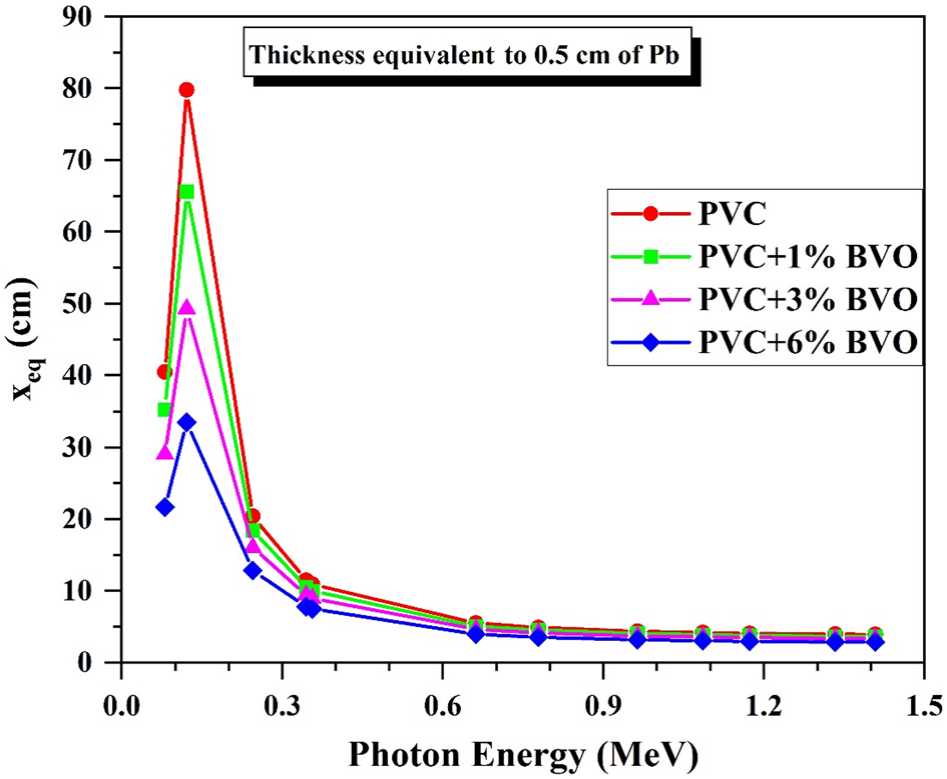
Variation of the equivalent thickness (X_eq_, cm) versus the incident gamma-ray energy.

The thickness equivalent (Xeq, cm) refers to the thickness of fabricated PVC + x% BVO nanocomposite films comparable to a pure lead sheet (ρ = 11.35 g/cm^3^) with a thickness of 0.5 cm^43^. Figure 16 illustrates the relationship between photon energies and Xeq values in PVC + x% BVO nanocomposite films. The X_eq_ values reduce as gamma-ray energy, and the percentage of BiVO_4_ nanofillers rises. Except at the absorption edges of Pb, which causes a rise in the LAC values of Pb compared to the LAC values of PVC + x% BVO in its energy region (0.081–0.1) MeV, growing X_eq_ values, as detected at 0.1218 MeV, reaching 79.821 cm for pure PVC and 33.515 cm for PVC + 6% BVO, as shown in Fig. 16. Then, as the incident gamma-ray energy values increased, X_eq_ started declining dramatically. The PVC + 6% BVO is reported to have the lowest X_eq_, with values ranging from 21.674 cm at 0.081 MeV to 2.866 cm at 1.408 MeV.

The measurement of effective atomic number (Z_eff_) and effective electron density (N_eff_) values are widely used in the exploration of the energy-dependent alterations of materials derived to develop alternative shielding against radiation products.

**Figure 17:**
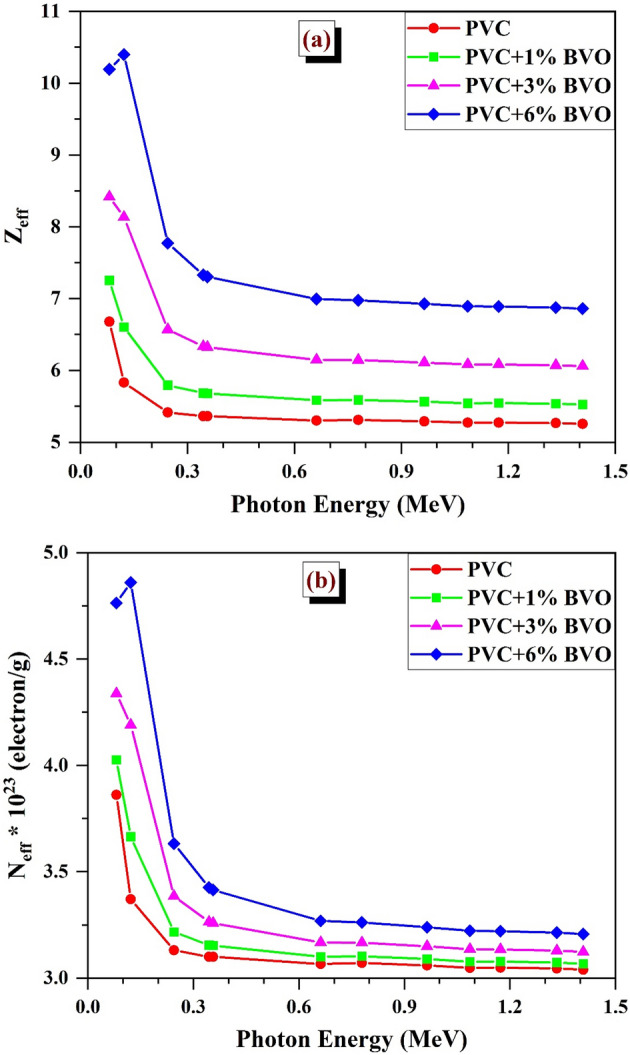
Variation of (**a**) Z_eff_ and (**b**) N_eff_ values for PVC + x% BVO nanocomposite films.

Determining the shielding characteristics of a multi-component composite as its equivalent element necessitates considering the effective atomic number (Z_eff_). The values of Z_eff_ gained are dependent upon the atomic number of the primary constituents present in the composition. Figure 17a, b depicts the Z_eff_ and N_eff_ variations of PVC + x% BVO nanocomposite films as a function of incident gamma-ray energy. As depicted in Fig. 17a, the effective atomic number (Z_eff)_ exhibits arise as the concentration of BiVO_4_ rises, which can be attributed to the incorporation of elements with high atomic numbers (Bi with Z = 83 and V with Z = 23). Moreover, the energy dependence of Z_eff_ exhibits similar characteristics to those marked for MAC^33^. The Z_eff_ tends to reach its maximum values for composites with low energy levels (< 0.2 MeV) due to the prominence of the photoelectric effect. It was observed that there was a rapid increase in Z_eff_ as the percentage of BiVO_4_ nanofiller boosted, which can be attributed to the presence of the K-absorption edge of the Bi atoms (90 keV). Significant observations have been established, ranging from 6.68 for PVC at Eγ = 0.081 MeV to 10.398 for PVC + 6%BVO at Eγ = 0.1218 MeV. A sharp decline was observed at intermediate energies due to the reduction in the photoelectric effect and the onset of Compton scattering interaction, leading to a reduction in Z_eff_ values up to 1.408 MeV. However, it was observed that the minimum values were present at energies between 0.2 MeV and above. The values exhibited slight variation and ranged from 5.305 to 6.995 for PVC and PVC + 6%BVO at Eγ = 0.6617 MeV, respectively.

Figure 17b illustrates the correlation between the BiVO_4_ content and the effective electron number (N_eff_) in PVC + x% BVO nanocomposite films. Like Z_eff_, N_eff_ also demonstrated a corresponding response to the rise in BVO concentration within the PVC polymer matrix, resulting in a significant increase in the effective Z and electron density of the produced nanocomposites. The highest N_eff_ was obtained as 3.861 × 10^23^ (electron/g) for PVC at the energy value of 0.081 MeV and 4.8599 × 10^23^ (electron/g) for PVC + 6%BVO at the energy value of 0.1218 MeV^31,33,44,45^.

## Conclusion

The present investigation utilizes PVC polymer composite as an alternative polymer matrix to develop environmentally sustainable polymer nanocomposites. The nanocomposites are doped with x% BiVO_4_ (BVO) nanofillers (where x = 0, 1, 3, and 6 wt.%) and are fabricated using the solution casting technique for gamma radiation shielding materials. The nanocomposite films composed of PVC and x% BVO were tested for characterization using various analytical techniques such as X-ray diffraction (XRD), high-resolution transmission electron microscopy (HR-TEM), Fourier transforms infrared spectroscopy (FTIR), scanning electron microscopy (SEM), and energy-dispersive X-ray spectroscopy (EDX). The composite samples exhibit a significant increase in the absorption coefficient and a decrease in the transmittance as the BiVO_4_ content rises. Also, the values obtained for PVC were 4.11 eV $({E}_{g}^{dir})$ and 4.142 eV ${(E}_{g}^{ind})$, while those for PVC + 6% BVO nanocomposite film were reduced to 4.01 eV ${(E}_{g}^{dir})$ and 4.06 eV ${(E}_{g}^{ind})$. The Phy-X/SPD theoretical program verified the Monte Carlo simulation code's (MCNP) simulated values; both results mutually agree. The LAC values exhibit a tendency to rise as the BVO ratio rises, with the lowest (highest) value of 0.3292 cm^−1^ (0.0783 cm^−1^) at 0.081 MeV (1.408 MeV) for pure PVC, and the highest (lowest) value of 0.6141 cm^−1^ (0.1073 cm^−1^) at 0.081 MeV (1.408 MeV) for PVC + 6% BVO nanocomposite film. The resistance and attenuation capacity of the manufactured PVC + x% BVO nanocomposites decreased with increasing photon energy and improved with rising BiVO_4_ content. At 0.081 MeV and 1.408 MeV, the transmission factors (TF) for PVC, PVC + 1% BVO, PVC + 3% BVO, and PVC + 6% BVO are approximately 71.97% and 92.47%, 70.97% and 92.17%, 68.97% and 91.55%, and 65.97% and 90.63%, respectively. Besides, at energy levels of 0.081 MeV and 1.408 MeV, the radiation protection efficiencies (RPE) with the highest and lowest values are approximately 28.03% and 7.53%, 29.03% and 7.83%, 31.03% and 8.45%, and 34.03% and 9.37% for PVC, PVC with 1% BVO, PVC with 3% BVO, and PVC with 6% BVO, respectively.These findings suggest that composition is more efficient in attenuating gamma rays. The research results suggest that the correlation between the photon and the material depends upon the material's atomic density and chemical composition. The results obtained from the study confirm that the PVC + x% BiVO_4_ nanocomposites that were successfully fabricated have potential applications in optical and radiation shielding.

## Data Availability

All data generated or analysed during this study are included in this published article.
